# The epigenetic clock and telomere length are independently associated with chronological age and mortality

**DOI:** 10.1093/ije/dyx233

**Published:** 2017-11-27

**Authors:** Riccardo E Marioni, Sarah E Harris, Sonia Shah, Allan F McRae, Thomas von Zglinicki, Carmen Martin-Ruiz, Naomi R Wray, Peter M Visscher, Ian J Deary

First published online: 13 April 2016, 45(2), 424–432, *Int J Epidemiol*, 2016, doi: https://doi.org/10.1093/ije/dyw041

In this article, the Hannum and Horvath age predictors were mislabelled in an analysis file. Therefore, the Hannum results in the paper are for the Horvath index and vice versa. This error does not affect the main conclusions and the effect sizes are very similar for both the Hannum and Horvath measures.

The results have been corrected.

We apologize for the oversight.

