# Supplementary material for: The epigenetic clock and telomere length are independently associated with chronological age and mortality
Source: Int J Epidemiol. 2016 Apr 13;45(2):424–32. doi: 10.1093/ije/dyw041 (PMC4864882; doi:10.1093/ije/dyw041)
Supplement: Supplementary Data [file dyw041_supp_files.pdf]

OXFORD UNIVERSITY PRESS | International Journal of Epidemiology

The epigenetic clock and telomere length are independently associated with chronological age and mortality

Journal:	*International Journal of Epidemiology*
Manuscript ID	IJE-2015-05-0645.R2
Manuscript Type:	Original Article
Date Submitted by the Author:	18-Nov-2015
Complete List of Authors:	Marioni, Riccardo; University of Edinburgh,<br>Harris, Sarah; University of Edinburgh,<br>Shah, Sonia; University of Queensland,<br>McRae, Allan; University of Queensland,<br>von Zglinicki, Thomas; University of Newcastle,<br>Martin-Ruiz, Carmen; University of Newcastle,<br>Wray, Naomi; University of Queensland,<br>Visscher, Peter; The University of Queensland, University of Queensland Diamantina Institute; The University of Queensland, Queensland Brain Institute<br>Deary, Ian; University of Edinburgh,
Key Words:	epigenetic clock, telomeres, ageing, longitudinal, mortality

SCHOLARONE™ Manuscripts

For Peer Review Only

The epigenetic clock and telomere length are independently associated with chronological age and mortality

Riccardo E. Marioni[1,2,3,*], Sarah E. Harris[2,3], Sonia Shah[1,4], Allan F. McRae[1,4], Thomas von Zglinicki[5], Carmen Martin-Ruiz[5], Naomi R. Wray[1], Peter M. Visscher[1,2,4], Ian J. Deary[2,6]

[1] Queensland Brain Institute, The University of Queensland, Brisbane 4072, Australia

[2] Centre for Cognitive Ageing and Cognitive Epidemiology, University of Edinburgh, Edinburgh, EH8 9JZ, UK

[3] Medical Genetics Section, Centre for Genomic and Experimental Medicine, Institute of Genetics and Molecular Medicine, University of Edinburgh, Edinburgh EH4 2XU, UK

[4] University of Queensland Diamantina Institute, Translational Research Institute, The University of Queensland, Brisbane 4072, QLD, Australia

[5] Institute for Ageing and Health, University of Newcastle, Newcastle, NE4 5PL, UK

[6] Department of Psychology, University of Edinburgh, Edinburgh, EH8 9JZ, UK

*** Corresponding Author:** Riccardo Marioni, riccardo.marioni@ed.ac.uk

For Review Only

Word Count:

Summary: 245

Main text: 3,642

Summary:

Background: Telomere length and DNA methylation have been proposed as biological clock measures that track chronological age. Whether they change in tandem, or contribute independently to the prediction of chronological age, is not known.

Methods: We address these points using data from two Scottish cohorts: the Lothian Birth Cohorts of 1921 (LBC1921) and 1936 (LBC1936). Telomere length and epigenetic clock estimates from DNA methylation were measured in 920 LBC1936 participants (ages 70, 73, and 76 years) and in 414 LBC1921 participants (ages 79, 87, and 90 years).

Results: The epigenetic clock changed over time at roughly the same rate as chronological age in both cohorts. Telomere length decreased at 48-67 base pairs per year on average. Weak, non-significant correlations were found between epigenetic clock estimates and telomere length. Telomere length explained 6.6% of the variance in age in LBC1921, the epigenetic clock explained 10.0%, and combined they explained 17.3% (all $P<1x10^{-7}$). Corresponding figures for the LBC1936 cohort were 14.3%, 11.7%, and 19.5% (all $P<1x10^{-12}$). In a combined cohorts analysis the respective estimates were 2.8%, 28.5%, and 29.5%. Also in a combined cohorts analysis a one standard deviation increase in baseline epigenetic age was linked to a 22% increased mortality risk ($P = 2.6x10^{-4}$) while, in the same model, a one standard deviation increase in baseline telomere length was independently linked to an 11% decreased mortality risk (P = 0.06).

Conclusions: These results suggest that telomere length and epigenetic clock estimates are independent predictors of chronological age and mortality risk.

Keywords: epigenetic clock; telomeres; ageing; longitudinal; mortality

Key messages:

For Review Only

Telomere length and DNA methylation are potential biological markers of ageing.

They do not correlate in later life between ages 70 and 90.

They associate independently with chronological age.

Both are predictors of mortality in an older population.

For Review Only

Introduction:

Biological clocks present an exciting opportunity to track and help understand ageing. For example, we can investigate differences in biological age for individuals of the same chronological age to see how environmental factors influence biological ageing rates. Numerous clocks have been proposed with varying accuracy in predicting chronological age. Two of the most promising clocks have been based on telomere length [1] and DNA methylation [2-4]. Telomeres are short sections of DNA and protein that cap the ends of chromosomes, protecting them from damage. They typically shorten over the life course, such that they have been used as biomarkers of ageing and age-related disease [5-7]. In the most recent systematic review, the correlation between telomere length and chronological age was reported to be around 0.3 [1]. Studies have described links between shorter telomere length and obesity [8], cancer [9, 10], diabetes [11], and cardiovascular disease [12]. There is also growing evidence to indicate that women have longer telomeres than men, although there remains some variability, particularly with regard to differing technologies and measurement issues [13]. To date, there have been few studies examining longitudinal telomere change. Typically these have been carried out in small samples and at only two time points [1, 14].

Biological markers of ageing have been derived from DNA methylation data. DNA methylation represents chemical modifications to the genome and is involved in the regulation of genes. In 2013, Hannum et al. used information from 71 CpG sites (measured in whole blood) to predict chronological age in 656 individuals [2]. They found a correlation of 0.96 between their measure and actual age. Other DNA methylation-based biological markers of ageing, also known as epigenetic clocks, have been derived using methylation data from multiple tissue sources [3]. Studies have linked faster-running epigenetic clocks to higher

For Review Only

mortality risk [15], and cross-sectionally to cognitive and physical health [16] but not with other health outcomes such as smoking, diabetes, heart disease, and genetic factors such as *APOE* [15].

In the present study we use longitudinal data on both telomere length and DNA methylation to determine if (i) the epigenetic clock correlates with telomere length, (ii) baseline epigenetic clock estimates associate with changes in telomere length, (iii) the epigenetic clock and telomere length correlate with age independently, (iv) the epigenetic clock and telomere length associate with mortality risk independently and (v) there are any epigenome-wide associations between telomere length and individual CpGs.

Methods:

Lothian Birth Cohorts

Data came from the Lothian Birth Cohorts of 1921 and 1936 (LBC1921 and LBC1936), which are longitudinal studies of ageing [17-19]. The cohorts contain individuals born in either 1921 or 1936 who participated in the Scottish Mental Surveys of 1932 or 1947, when nearly all 11-year-old children sat a cognitive test. Most of the participants were living in the City of Edinburgh or its surrounding (Lothians) area of Scotland in later life when the studies commenced. 550 LBC1921 participants were recruited at mean age 79 and have completed up to five clinical examinations at mean ages 79, 83, 87, 90, and 92. In LBC1936, 1,091 participants were recruited at age 70 and have completed up to three clinical examinations at mean ages 70, 73, and 76. In both cohorts detailed information has been obtained on lifestyle and health outcomes, genetics, and epigenetics.

LBC consent

For Review Only

Following written informed consent, venesected whole blood was collected for DNA extraction in both LBC1921 and LBC1936. Ethics permission for the LBC1921 was obtained from the Lothian Research Ethics Committee (Wave 1: LREC/1998/4/183; Wave 3:1702/98/4/183) and the Scotland A Research Ethics Committee (Wave 4:10/S1103/6). Ethics permission for the LBC1936 was obtained from the Multi-Centre Research Ethics Committee for Scotland (Wave 1: MREC/01/0/56), the Lothian Research Ethics Committee (Wave 1: LREC/2003/2/29) , and the Scotland A Research Ethics Committee (Waves 2 and 3: 07/MRE00/58).

For Review Only

DNA methylation

A detailed protocol describing DNA measurement in the Lothian Birth Cohorts has been presented previously [20]. Briefly, methylation measurements from whole blood were taken from LBC1936 participants at waves 1 (age 70, n=1,004), 2 (age 73, sub-group n = 336), and 3 (age 76, sub-group n = 332) and from LBC1921 participants at waves 1 (age 79, n=517), 3 (age 87, n=181), and 4 (age 90, n=87). The Illumina HumanMethylation450 BeadChips array was used to measure DNA methylation at 485,512 sites. Quality control was carried out on these data to remove (i) probes with a low detection rate (ii) low quality samples (iii) samples with a low call rate (iv) samples where there was a sex mismatch. Post-QC there were 450,726 autosomal probes available for analysis in 920, 299, and 273 participants at the three LBC1936 waves, respectively, and in 446, 175, and 82 participants at the three LBC1921 waves, respectively (Table 1). Seventy-one of these probes were used to calculate DNA methylation age using the regression weights supplied by Hannum et al [2]. Hereafter, we refer to this quantity as Hannum age or Hannum epigenetic clock estimates. For the epigenome-wide association studies, beta values were corrected for effects of sample plate,

BeadChip, position on BeadChip, and hybridization date using a generalized linear model with a logistic link function. Residuals from this model were used in further analyses.

Telomere measurement

Telomere length was measured at the same waves as methylation in both LBC cohorts (LBC1921 at waves 1, 3, and 4, and in LBC1936 at waves 1, 2, and 3; Table 1) using a quantitative real-time polymerase chain reaction (PCR) assay [21]. Four internal control DNA samples were run within each plate to correct for plate-to-plate variation. PCRs were performed on an Applied Biosystems (Pleasonton, CA, USA) 7900HT Fast Real Time PCR machine.

Mortality ascertainment

For both LBC1921 and LBC1936, mortality status was obtained via data linkage from the National Health Service Central Register, provided by the General Register Office for Scotland (now National Records of Scotland). Participant deaths and cause of death are routinely flagged to the research team on approximately a 12-weekly basis.

Statistical Analyses

Pearson correlations were calculated between the Hannum epigenetic clock estimates (Hannum age) and telomere length at each wave in both LBC1921 and LBC1936. Linear mixed models were used to examine independently the longitudinal changes in telomere length and Hannum age [22]. Sex was included as a covariate, age as the timescale, and participant as a random effect on the intercept. Across all of our mixed effects models there was no statistical evidence for the inclusion of a person-specific random slope parameter. Interaction terms were included to test whether men and women changed at different rates. A

For Review Only

mixed model (with age and sex as fixed effects, and a participant random effect on the intercept) was used to examine the change in telomere length that was predicted by baseline epigenetic age acceleration (residuals from a linear regression of Hannum age on chronological age); the coefficient of interest in this model was the interaction term between baseline age acceleration and chronological age. Interactions between sex and chronological age were also investigated. With age as the dependent variable, linear mixed effects models were used to model the longitudinal change in chronological age as a function of telomere length and Hannum age. We first examined the variables independently and then together in the same model. To obtain approximate R^2 estimates for the respective models, we re-ran the analysis using linear regression on a randomly sampled subset of the data whereby each individual was allowed to provide information to only a single wave. Finally, we combined the two cohorts into a single sample and re-ran the age prediction analyses described above. For the survival (time-to-death) analyses, Cox proportional hazards models were built using baseline data on age, telomere length, and Hannum age as predictors along with sex. An epigenome-wide association study was performed in both LBC1921 and LBC1936 using telomere data from all waves. Linear mixed models were run with the CpG probes as the dependent variable and telomere length as the independent variable of interest; covariates included age, sex, estimated white blood cell counts [23] as fixed effects, and individual participant ID as a random effect. A standard error-based meta analysis of the EWAS results was performed in METAL [24]. All other analyses were conducted in R, utilising the "lme4", "lmerTest", and "survival" libraries [25-28].

Results:

A summary of the Lothian Birth Cohort data is presented in Table 1 with a breakdown of the sample by number of waves completed presented in Supplementary Tables 1 and 2. The

For Review Only

small standard deviations for age emphasise that all participants (in the respective cohorts) were born in the same year. There is modest attrition (27% of the sample) over time in the younger 1936 cohort with a much greater loss of individuals over the longer period in the older cohort (77% of the sample); most of the dropout is due to mortality as shown in the Cox models in Supplementary Table 3. Individuals with shorter telomeres at baseline were more likely to drop out of LBC1921 although there was little difference in baseline telomere length and dropout in LBC1936 (Supplementary Tables 1 and 2). The gender balance remains consistent in both cohorts over time. Hannum age point estimates are, on average, slightly lower than chronological age but show a mean increase over time; average telomere length shortens over time.

Correlations between telomere length and the epigenetic clock

The correlations between telomere length and Hannum age are reported in Table 2. There were no significant associations or consistent direction of association between the measures at any of the waves in either of the cohorts. The correlation coefficients were small, not exceeding -0.047 (SE = 0.06) in LBC1936. In the smaller LBC1921, the largest correlation was 0.12, which carried a standard error of 0.09.

Change in telomere length and the epigenetic clock over time

Both telomere length and Hannum age changed over time in the two cohorts (Figure 1). In LBC1936, telomere length decreased over the six year follow-up by an average of 0.098 (SE 0.005, $P<2x10^{-16}$) standard deviations (the standard deviation was derived using telomere data from across all waves; the decline is equivalent to a drop of 67 base pairs) per year. Hannum age increased by an average of 0.14 (SE 0.007, $P<2x10^{-16}$) standard deviations per year, which is equivalent to 0.96 epigenetic clock years per calendar year. Similar

For Review Only

associations were seen in LBC1921 where telomere length decreased by 0.083 (SE 0.008, $P<2x10^{-16}$) standard deviations (equivalent to 48 base pairs) per year on average, and Hannum age increased by 0.085 (SE 0.006, $P<2x10^{-16}$) standard deviations per year (0.60 years per calendar year) on average. In LBC1936 but not LBC1921 there was evidence for an interaction between rate of decline in telomere length and sex. Men declined faster by an average of 0.04 SDs (~29 base pairs, $P=5.9x10^{-6}$) per year. In a pooled analysis of both cohorts, telomere length declined at an average of 37 (SE 2, $P<2x10^{-16}$) base pairs per year while Hannum age increased by 0.76 years per calendar year (SE 0.02, $P<2x10^{-16}$). There was an interaction between gender and rate of telomere change - men declined faster by an average of 10 (SE 4, P=0.017) base pairs per year.

Baseline methylation age acceleration (defined as the residuals from the regression of Hannum age on chronological age) did not predict longitudinal decline in telomere length in LBC1921 (beta = $4.8x10^{-3}$, SE = $8.9x10^{-3}$, P = 0.59), LBC1936 (beta = $-3.5x10^{-3}$, SE = $4.9x10^{-3}$, P = 0.48), or in a pooled analysis (beta = $-5.7x10^{-4}$, SE = $3.6x10^{-3}$, P = 0.88).

Telomere length and the epigenetic clock as predictors of age

The LBC1936 linear regression models with age as the dependent variable yielded R^2 coefficients of 6.6% for telomere length, 10.0% for Hannum age, and 17.3% for the model with both (all $P<1x10^{-12}$). The corresponding estimates in LBC1921 were 14.3% for telomere length and 11.7% for Hannum age. In the model with both predictors, the R^2 estimate increased to 19.5% (all $P<1x10^{-7}$). The results for both LBC cohorts are presented in Figure 2. In the combined cohorts analysis, the R^2 coefficients for telomere length and Hannum age were 2.8% and 28.5%, respectively, with 29.5% of the variance explained in the model with both predictors. Supplementary Table 4 shows the regression coefficients for the linear

For Review Only

model where each participant contributed to no more than one time point, and the linear mixed model that included ID as a random effect.

Telomere length and the epigenetic clock as predictors of mortality

The results from the survival analyses for all-cause mortality are reported in Table 3. In a baseline-age, and sex adjusted model in LBC1921, longer baseline telomere length was associated with a decreased mortality risk - hazard ratio = 0.86 (per standard deviation change; and 95% confidence interval = 0.75, 0.97, P=0.015) - while a higher Hannum age was associated with an increased mortality risk - 1.22 [1.08, 1.39], P=0.001 (also per standard deviation change). In LBC1936 the corresponding estimates were 0.96 [0.80, 1.14], P=0.63 for telomere length and 1.18 [1.01, 1.38], P=0.040 for Hannum age. For both cohorts the Hannum age associations with mortality have been reported previously [16]. However, these associations were independent of telomere length. In a pooled cohorts analysis an older Hannum age was associated with an increased mortality risk (HR 1.22 [1.10, 1.37], P = 2.6×10^{-4}); there was a borderline-significant protective effect of increased telomere length (HR 0.89 [0.80, 1.01], P = 0.06). The all-cause mortality associations for telomere length and Hannum age without mutual adjustment are reported in Supplementary Table 3.

Replication with the Horvath methylation age predictor

Re-running the above analyses with the Horvath predictor [3] in place of the Hannum measure yielded near identical findings. The Horvath measure correlated between -0.08 and 0.19 with telomere length. The latter correlation was statistically significant (P=0.031) although this was at wave 3 of LBC1921 where the sample size is small (n=131). Baseline Horvath age acceleration did not predict longitudinal change in telomere length (standardised beta 1.5×10^{-3}, SE 3.8×10^{-3}, P=0.70). In the age prediction models, both Horvath age and

For Review Only

telomere length were independently associated with age - the effect sizes for telomere length are very similar to those from the Hannum models (results not shown). Similar independent findings are observed in the mortality survival models and the estimates map neatly to those from the Hannum models (results not shown).

Epigenome-wide association study of telomere length

The epigenome-wide association study analysis revealed a single hit in both cohorts after a Bonferroni correction for multiple testing (Supplementary Figure 1). Telomere length was associated with cg20489909 ($P=7.6x10^{-8}$) on chromosome 2 in LBC1921 and cg11606261 ($P=2.5x10^{-8}$) on chromosome 12 in LBC1936. The genomic inflation statistics for the two models were 0.93 and 1.02, respectively. The LBC1921 hit was not significant in LBC1936 (P=0.27) while the LBC1936 hit was nominally (P<0.05) significant in LBC1921 (P=0.005) but with an effect size in the opposite direction. In a meta-analysis of the results there were no epigenome-wide significant hits (Supplementary Figure 1).

Discussion

These analyses find that telomere length and epigenetic clock estimates are uncorrelated in later life and that they are independent predictors of chronological age, in similar magnitude. The epigenetic clock estimates and telomere length are both associated independently with mortality risk in LBC1921; only the epigenetic clock estimates are associated with mortality in LBC1936. Where significant, the effect sizes are in the expected directions - shorter telomeres and a faster running epigenetic clock (older epigenetic age relative to those of a similar chronological age) are associated with an increased mortality risk. There were no individual CpG associations with telomere length in the EWAS meta-analysis.

For Review Only

These findings are important for ageing research as they point towards different pathways/mechanisms that are being tagged by telomeres and the epigenetic clock. What these might be is beyond the scope of the current study. In terms of the disease-related and health outcome-related pathways, larger studies and collaborative efforts will be required to link both telomere length and the epigenetic clock to such outcomes. For example, in a study of the epigenetic clock and mortality using data from four cohorts, no consistent associations were found between it and measures such as education, type 2 diabetes, hypertension, APOE status, smoking, cardiovascular disease [15]. There were consistent sex differences with men having a faster running clock [3, 15]. Previous research using LBC1936 data has also shown cross-sectional associations between epigenetic clock estimates and cognitive ability and physical fitness (grip strength and lung function, but not walking speed) [16]. There is also evidence to link accelerated epigenetic ageing in those with Down Syndrome [29], in addition to a faster running epigenetic clock in the livers of obese individuals [30]. Previous studies of telomeres have linked shorter length to obesity, cardiovascular disease, and diabetes, but not to physical or cognitive decline [8-12, 31].

For Review Only

In the present study, we focussed on the Hannum epigenetic clock [2] in favour of the multi-tissue derived clock built by Horvath [3] due to the same tissue (with the same white blood cell confounders) being relevant to both the Hannum clock and the telomere lengths. However, re-running the models with the Horvath predictor yielded near identical findings. One peculiarity of our data is the "increase" in telomere length between ages 79 and 87 in LBC1921 (Supplementary Table 2). However, upon the exclusion of individuals not present at both waves, where dropout was primarily due to death, the mean telomere lengths appears to be stable rather than increasing (Supplementary Table 2). Similarly, we also observe longer telomeres in the LBC1921 participants at age 79 compared to the LBC1936

participants at age 76 (Table 1 and Supplementary Tables 1 and 2). This remains the case when we consider LBC1921 participants who dropped out after the first wave of data collection. Possible explanations for this include cohort effects, whereby those recruited at age 79 are healthier (have longer telomeres, in this instance) than those recruited at age 70 and who survive to age 76. Another explanation could be batch effects of the assay however, all samples were processed identically and at the same time. However, a recent study of telomere length and age in over 100,000 individuals suggests that telomere length declines over mid-life but remains stable/subtly increases in later life [32]. Nonetheless, measurement error and subtle batch effects in the telomere measures may have undermined the associations between it and methylation age. We also observe a sizable mean difference between the Hannum epigenetic age and chronological age, although, as previously reported [15], this intercept effect is not overly concerning given the birth cohort data that we are investigating. Moreover, the longitudinal changes in the epigenetic clock estimates occur at the same rate as chronological ageing in LBC1936, which gives us confidence that they are tracking the process well. The main limitation of the study is the relatively small sample size, particularly for LBC1921 due to dropout for death and sub-group methylation typing at waves 2 and 3 in LBC1936. However, very few datasets exist with both longitudinal (three wave) telomere length and DNA methylation, particularly in this age range. A more complete summary of the change in telomere length over time in both LBC studies (including LBC1921 data at wave 5, age 92) is currently in preparation, along with concurrent changes in physical and cognitive fitness.

The integration of the joint telomere-epigenetic clock findings with the current literature is difficult as, to our knowledge, this is the first time that such results have been reported. The mortality results for the epigenetic clock have been previously reported in both LBC1921 and

For Review Only

LBC1936 and are consistent with those found in two other independent cohorts [15]. The first telomere-mortality findings described a near 2-fold increased risk for those with short compared to long telomeres, along with increased risks for some cause-specific mortality outcomes [33]. When considered as a continuous variable, telomere length remained linked to an excess all-cause mortality risk [33]. A much larger study (n=64,637, ndeaths=7,607) confirms these findings, reporting an increased all-cause mortality risk of 40% for those in the decile of longest compared to shortest telomeres [34].

For Review Only

The cohorts' narrow age range at each time point and the follow-up period of 13 and 6 years, respectively, means that our R^2 estimates for predicting chronological age are likely to be smaller than those obtained from cohorts with a wider range of ages. This is particularly the case for the epigenetic clock estimates, which typically yield correlations of above 0.8 or 0.9 in samples with age heterogeneity [2-4, 15]. Indeed, we observed an increased R^2 for the epigenetic clock estimates when we combined the two LBC studies into a single cohort. This pooled analysis suggested that methylation age is a much better predictor of chronological age (in old age) than telomere length. Whether both the independence and the magnitudes of the biological ageing estimates to predict chronological age remain consistent in younger populations, where telomere length is likely to be more effective in predicting age [32], is unknown but of great interest. Another point that needs to be considered in future studies is tissue specificity. Here, we measured telomere length and DNA methylation in blood. Whether similar findings are seen in other tissues is not known.

This study shows, for the first time, the relationship between two biological markers of ageing: telomere length and the DNA methylation-based epigenetic clock. In our data, both associate independently with chronological age. The measures do not correlate with each

other nor do differences in baseline epigenetic clock measures predict change in telomere length. Further research is warranted, using datasets with more events/deaths, to robustly assess the joint relationship between the two biological markers of ageing and mortality. In summary, telomere length and DNA methylation-based epigenetic clock estimates appear to be tagging two different aspects of the ageing process.

Acknowledgements

We thank the cohort participants and team members who contributed to these studies. This work was supported by multiple sources. Phenotype collection in the Lothian Birth Cohort 1921 was supported by the UK's Biotechnology and Biological Sciences Research Council (BBSRC), The Royal Society and The Chief Scientist Office of the Scottish Government. Phenotype collection in the Lothian Birth Cohort 1936 was supported by Age UK (The Disconnected Mind project). Methylation typing was supported by Centre for Cognitive Ageing and Cognitive Epidemiology (Pilot Fund award), Age UK, The Wellcome Trust Institutional Strategic Support Fund, The University of Edinburgh, and The University of Queensland. REM, SEH, IJD and PMV are members of the University of Edinburgh Centre for Cognitive Ageing and Cognitive Epidemiology (CCACE). CCACE is supported by funding from the BBSRC, the Medical Research Council (MRC), and the University of Edinburgh as part of the cross-council Lifelong Health and Wellbeing initiative (MR/K026992/1). CMR is supported by the NIHR Biomedical Research Centre at Newcastle upon Tyne Hospitals NHS Foundation Trust and Newcastle University.

Data Accession

The European Genome-Phenome accession number for the LBC methylation data reported in this paper is EGAS00001000910.

For Review Only

References

1. Müezzinler A, Zaineddin AK, Brenner H. A systematic review of leukocyte telomere length and age in adults. Ageing Res. Rev., 2013; 12(2): 509-519.

2. Hannum G, Guinney J, Zhao L, Zhang L, Hughes G, Sadda S, et al. Genome-wide methylation profiles reveal quantitative views of human aging rates. Mol. Cell, 2013; 49(2): 359-367.

3. Horvath S. DNA methylation age of human tissues and cell types. Genome Biol., 2013: 14(10); R115

4. Bocklandt, S, Lin, W, Sehl, ME, Sánchez, FJ, Sinsheimer, JS, Horvath, S, et al. Epigenetic predictor of age. PLoS ONE, 2011: 6(6): e14821.

5. Hunt SC, Chen W, Gardner JP, Kimura M, Srinivasan SR, Eckfeldt JH, et al. Leukocyte telomeres are longer in African Americans than in whites: the National Heart, Lung, and Blood Institute Family Heart Study and the Bogalusa Heart Study. Aging Cell, 2008; 7(4): 451-458.

6. Sanders JL, Newman AB. Telomere length in epidemiology: a biomarker of aging, age-related disease, both, or neither? Epidemiol Rev, 2013; 35: 112-131.

7. von Zglinicki T, Martin-Ruiz CM. Telomeres as biomarkers for ageing and age-related diseases. Curr. Mol. Med., 2005; 5(2): 197-203.

8. Njajou OT, Cawthon RM, Blackburn EH, Harris TB, Li R, Sanders JL, et al. Shorter telomeres are associated with obesity and weight gain in the elderly. Int J Obes (Lond), 2012; 36(9): 1176-1179.

9. Wentzensen IM, Mirabello L, Pfeiffer RM, Savage SA. The association of telomere length and cancer: a meta-analysis. Cancer Epidemiol. Biomarkers Prev., 2011; 20(6): 1238-1250.

For Review Only

10. Ma H, Zhou Z, Wei S, Liu Z, Pooley KA, Dunning AM, et al. Shortened telomere length is associated with increased risk of cancer: a meta-analysis. PLoS ONE, 2011; 6(6): e20466.

11. Zhao J, Miao K, Wang H, Ding H, Wang DW. Association between telomere length and type 2 diabetes mellitus: a meta-analysis. PLoS ONE, 2013; 8(11): e79993.

12. Haycock PC, Heydon EE, Kaptoge S, Butterworth AS, Thompson A, Willeit P. Leucocyte telomere length and risk of cardiovascular disease: systematic review and meta-analysis. BMJ, 2014; 349: g4227.

13. Gardner M, Bann D, Wiley L, Cooper R, Hardy R, Nitsch D, et al. Gender and telomere length: systematic review and meta-analysis. Exp. Gerontol., 2014; 51: 15-27.

14. Gardner MP, Martin-Ruiz C, Cooper R, Hardy R, Sayer AA, Cooper C, et al. Telomere length and physical performance at older ages: an individual participant meta-analysis. PLoS ONE, 2013; 8(7): e69526.

15. Marioni RE, Shah S, McRae AF, Chen BH, Colicino E, Harris SE, et al. DNA methylation age of blood predicts all-cause mortality in later life. Genome Biol., 2015; 16(1): 25.

16. Marioni RE, Shah S, McRae AF, Ritchie SJ, Muniz-Terrera G, Harris SE, et al. The epigenetic clock is correlated with physical and cognitive fitness in the Lothian Birth Cohort 1936. Int J Epidemiol, 2015; Epub ahead of print.

17. Deary IJ, Whiteman MC, Starr JM, Whalley LJ, Fox HC. The impact of childhood intelligence on later life: following up the Scottish mental surveys of 1932 and 1947. J Pers Soc Psychol, 2004; 86(1): 130-147.

18. Deary IJ, Gow AJ, Taylor MD, Corley J, Brett C, Wilson V, et al. The Lothian Birth Cohort 1936: a study to examine influences on cognitive ageing from age 11 to age 70 and beyond. BMC Geriatr, 2007; 7: 28.

For Review Only

19. Deary IJ, Gow AJ, Pattie A, Starr JM. Cohort profile: the Lothian Birth Cohorts of 1921 and 1936. Int J Epidemiol, 2012; 41(6): 1576-1584.

20. Shah S, McRae AF, Marioni RE, Harris SE, Gibson J, Henders AK, et al. Genetic and environmental exposures constrain epigenetic drift over the human life course. Genome Res., 2014; 24(11): 1725-1733.

21. Martin-Ruiz C, Saretzki G, Petrie J, Ladhoff J, Jeyapalan J, Wei W, et al. Stochastic variation in telomere shortening rate causes heterogeneity of human fibroblast replicative life span. J. Biol. Chem., 2004; 279(17): 17826-17833

22. Laird NM, Ware JH. Random-effects models for longitudinal data. Biometrics, 1982; 38(4): 963-74.

23. Houseman EA, Accomando WP, Koestler DC, Christensen BC, Marsit CJ, Nelson HH, et al. DNA methylation arrays as surrogate measures of cell mixture distribution. BMC Bioinformatics, 2012; 13: 86.

24. Willer CJ, Li Y, Abecasis GR. METAL: fast and efficient meta-analysis of genomewide association scans. Bioinformatics, 2010; 26(17): 2190-2191.

25. R Core Team. R: A Language and Environment for Statistical Computing, 2013.

26. Kuznetsova A, Brockhoff PB, Christensen RHB. lmerTest: Tests for random and fixed effects for linear mixed effect. R package version 2.0-0, 2013.

27. Bates D, Maechler M, Bolker B, and Walker S. lme4: Linear mixed-effects models using Eigen and S4. R package version 1.0-4, 2013.

28. Therneau, TM. A Package for Survival Analysis in S, 2013.

29. Horvath S, Garagnani P, Bacalini MG, Pirazzini C, Salvioli S, Gentilini D, et al. Accelerated epigenetic aging in Down syndrome. Aging Cell, 2015; Epub ahead of print.

For Review Only

30/19. Horvath S, Erhart W, Brosch M, Ammerpohl O, von Schönfels W, Ahrens M, et al. Obesity accelerates epigenetic aging of human liver. Proc. Natl. Acad. Sci. U.S.A., 2014; 111(43): 15538-15543.

31. Harris SE, Deary IJ, MacIntyre A, Lamb KJ, Radhakrishnan K, Starr JM, et al. The association between telomere length, physical health, cognitive ageing, and mortality in non-demented older people. Neurosci. Lett., 2006; 406(3): 260-264.

32. Lapham K, Kvale MN, Lin J, Connell S, Croen LA, Dispensa BP, et al. Automated Assay of Telomere Length Measurement and Informatics for 100,000 Subjects in the Genetic Epidemiology Research on Adult Health and Aging (GERA) Cohort. Genetics, 2015; E-Pub ahead of print.

33. Cawthon RM, Smith KR, O'Brien E, Sivatchenko A, Kerber RA. Association between telomere length in blood and mortality in people aged 60 years or older. Lancet, 2003; 361(9355): 393-395.

For Review Only

Table 1: Summary descriptive data for the Lothian Birth Cohorts of 1921 and 1936. mean (SD) or n (%)

LBC1936

Wave	n	Age (yrs)	Sex (F)	Telomere Length (bp)	$N_{methylation}$	Hannum Methylation Age (yrs)
1	1,070	69.5 (0.8)	530 (50)	4201 (560)	920	65.9 (6.5)
2	853	72.5 (0.7)	409 (48)	3966 (738)*	299	67.2 (6.7)
3	691	76.2 (0.7)	331 (48)	3739 (686)	273	71.5 (6.1)

LBC1921

Wave	n	Age (yrs)	Sex (F)	Telomere Length (bp)	$N_{methylation}$	Hannum Methylation Age (yrs)
1	529	79.1 (0.6)	308 (58)	4089 (413)**	446	73.7 (7.0)
3	189	86.6 (0.4)	101 (53)	4209 (564)†	174	77.7 (5.9)
4	92	90.1 (0.1)	48 (52)	3179 (722)	82	79.3 (6.1)

* 9 samples missing ** 32 samples missing † 43 samples missing

For Review Only

Table 2: Pearson correlations between Hannum methylation age and telomere length.

	n	r (SE*)	P-Value
LBC1936			
Wave 1 (age 70yrs)	920	-0.019 (0.033)	0.56
Wave 2 (age 73yrs)	290	0.011 (0.059)	0.85
Wave 3 (age 76yrs)	273	-0.047 (0.061)	0.44
LBC1921			
Wave 1 (age 79yrs)	414	0.020 (0.049)	0.68
Wave 3 (age 87yrs)	131	0.117 (0.087)	0.18
Wave 4 (age 90yrs)	82	0.062 (0.111)	0.58

* Approximate standard errors were calculated using the formula $\sqrt{(1-r^2)/(n-2)}$

For Review Only

Table 3: Mortality survival model output for Hannum methylation age and telomere length.

LBC1936 (n = 920, n_{events} = 135)

Covariate	Hazard Ratio	95% CI	P-Value
Age (years)	1.04	[0.84, 1.29]	0.69
Sex (Female)	0.64	[0.45, 0.92]	0.016
Telomere Length	0.96	[0.81, 1.14]	0.64
Hannum age	1.18	[1.01, 1.39]	0.036

LBC1921 (n = 414, n_{events} = 280)

Covariate	Hazard Ratio	95% CI	P-Value
Age (years)	0.97	[0.78, 1.20]	0.75
Sex (Female)	0.86	[0.67, 1.10]	0.22
Telomere Length	0.87	[0.76, 0.97]	0.013
Hannum age	1.22	[1.08, 1.38]	0.001

LBC1921 + LBC1936 (n = 1,344, n_{events} = 415)

Covariate	Hazard Ratio	95% CI	P-Value
Age (years)	1.12	[1.09, 1.15]	$<2x10^{-16}$
Sex (Female)	0.79	[0.64, 0.97]	0.02
Telomere Length	0.89	[0.80, 1.01]	0.06
Hannum age	1.22	[1.10, 1.37]	$2.6x10^{-4}$

Effect sizes for telomere length and Hannum age are per standard deviation.

For Review Only

Supplementary Table 1: LBC1936 descriptive data on complete cases split by number of waves attended. mean (SD) or n (%)

Wave	n visits	n	Age (yrs)	Sex (F)	Telomere Length (bp)	Hannum Age (yrs)
1	1	624	69.8 (0.8)	315 (50)	4200 (579)	66.5 (6.3)
	2	46	69.1 (0.6)	18 (39)	4240 (614)	64.7 (9.6)
	3	219	69.0 (0.6)	112 (51)	4176 (491)	64.6 (6.3)
2	2	46	72.1 (0.5)	18 (39)	4224 (1192)	67.4 (6.8)
	3	219	72.0 (0.5)	112 (51)	3909 (681)	69.1 (6.5)
3	3	219	75.8 (0.5)	112 (51)	3663 (678)	71.4 (6.2)

6 individuals had one visit at wave 2; 4 individuals had one visit at wave 3; 21 individuals had 2 visits, one of which was at wave 3.

For Review Only

Supplementary Table 2: LBC1921 descriptive data on complete cases split by number of waves attended. mean (SD) or n (%)

Wave	n visits	n	Age (yrs)	Sex (F)	Telomere Length (bp)	Hannum Age (yrs)
1	1	299	79.1 (0.6)	188 (63)	4064 (385)	74.3 (7.3)
	2	61	79.2 (0.6)	33 (54)	4110 (457)	72.6 (5.5)
	3	50	79.2 (0.6)	26 (52)	4231 (335)	74.2 (6.3)
3	2	61	86.7 (0.4)	33 (54)	4205 (571)	77.9 (5.1)
	3	50	86.7 (0.4)	26 (52)	4240 (353)	78.4 (6.6)
4	3	50	90.1 (0.1)	26 (52)	3194 (659)	80.2 (6.4)

10 individuals had one visit at wave 3; 18 individuals had one visit at wave 4; 14 individuals had two visits, one of which was at wave 4.

For Review Only

Supplementary Table 3: Survival model output for age- and sex-adjusted methylation age and telomere length.

LBC1936 (n = 920, n_{events} = 135)

Covariate	Hazard Ratio	95% CI	P-Value
Telomere Length	0.92	[0.78, 1.09]	0.34
Hannum age	1.18	[1.01, 1.39]	0.036

LBC1921 (n = 414, n_{events} = 280)

Covariate	Hazard Ratio	95% CI	P-Value
Telomere Length	0.86	[0.76, 0.97]	0.017
Hannum age	1.25	[1.11, 1.41]	$3x10^{-4}$

LBC1921 + LBC1936 ($n_{telo/Hannum}$ = 1,484/1,366, n_{events} = 437/427)

Covariate	Hazard Ratio	95% CI	P-Value
Telomere Length	0.88	[0.79, 0.99]	0.027
Hannum age	1.25	[1.12, 1.39]	$4x10^{-4}$

Effect sizes for telomere length and Hannum age are estimated in independent models and are reported per standard deviation.

For Review Only

Supplementary Table 4: Regression output for the age prediction models. The linear model takes a random sample of the population at each wave, such that each individual can only be included at a single wave. The linear mixed model includes a random intercept for participant ID to account for the clustering. The top half of the table shows the univariate results, the bottom half shows the multivariate results. All models adjust for sex.

Univariate Output		LBC1921		LBC1936		LBC1921 + LBC1936	
		Std. Beta	SE	Std. Beta	SE	Std. Beta	SE
Linear model (random subset)	Telomere	-0.37	0.06	-0.26	0.03	-0.15	0.03
	Hannum age	0.33	0.06	0.28	0.04	0.58	0.03
Linear mixed model	Telomere	-0.33	0.03	-0.25	0.02	-0.28	0.01
	Hannum age	0.31	0.03	0.29	0.02	0.59	0.02

Multivariate Output		LBC1921		LBC1936		LBC1921 + LBC1936	
		Std. Beta	SE	Std. Beta	SE	Std. Beta	SE
Linear model (random subset)	Telomere	-0.31	0.07	-0.25	0.04	-0.14	0.04
	Hannum age	0.30	0.06	0.26	0.04	0.55	0.03
Linear mixed model	Telomere	-0.33	0.03	-0.21	0.02	-0.21	0.02
	Hannum age	0.29	0.03	0.27	0.02	0.55	0.02

For Review Only

Figure 1: Spaghetti plots for change in Hannum age and telomere length over time in LBC1936 and LBC1921.

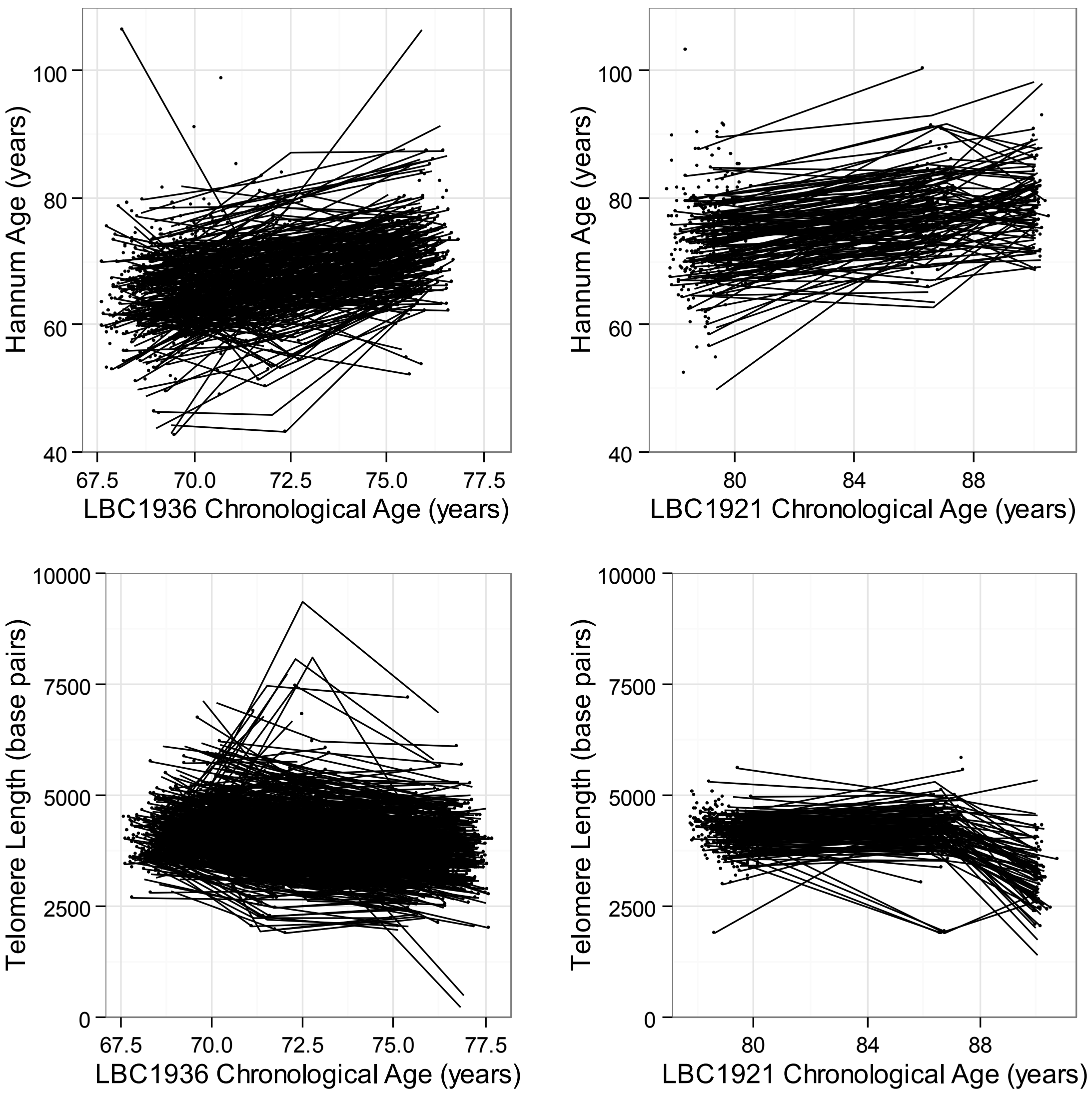

Figure 2: Proportion of variance in age explained by Hannum age and telomere length.

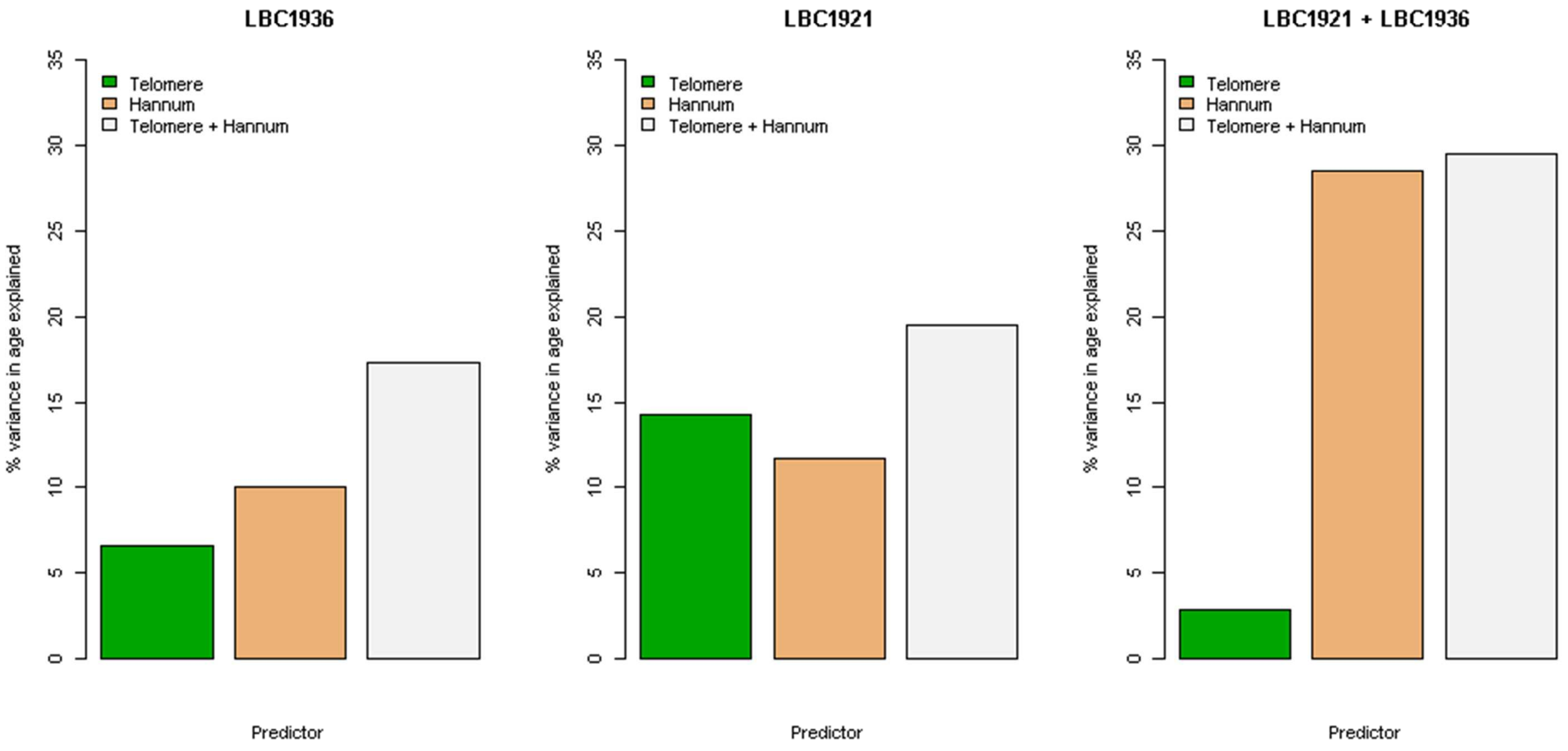

Supplementary Figure 1: EWAS of Telomere Length in LBC1921, LBC1936, and meta analysis.

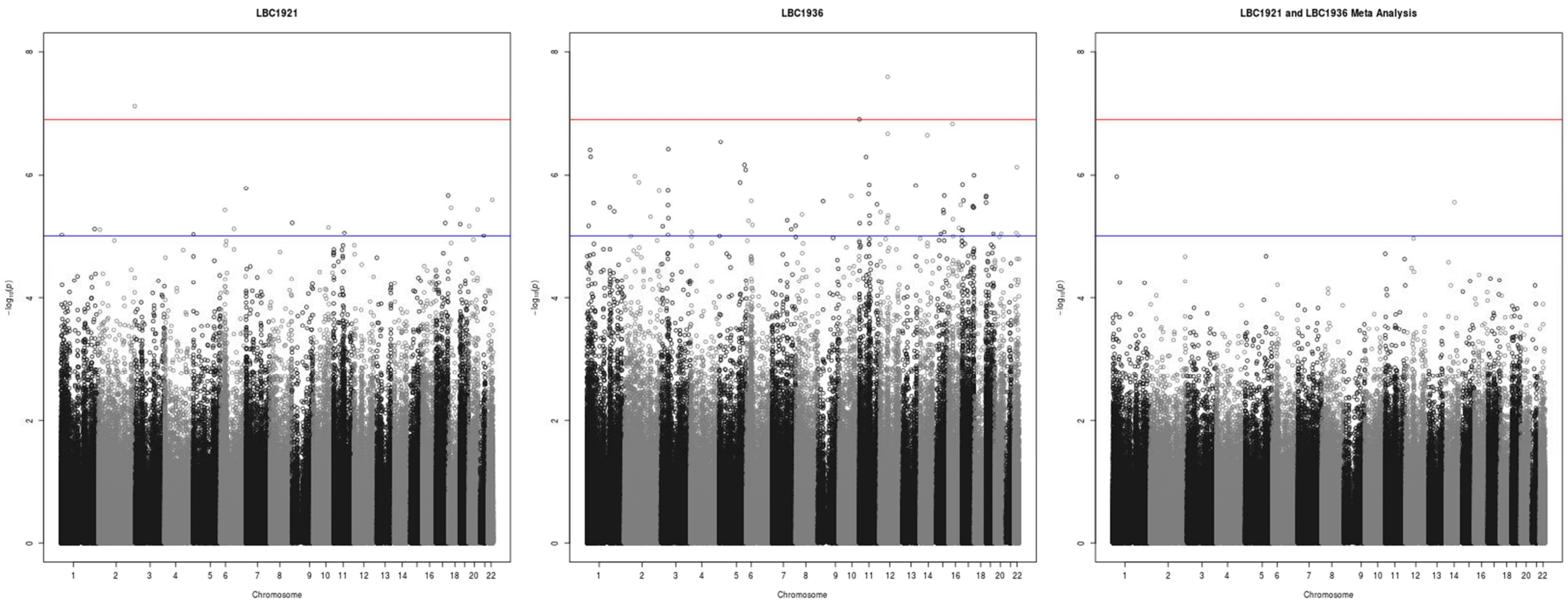

The red line represents the Bonferroni significant threshold of $P = 1.2\text{x}10^{-7}$; the blue line represents the nominal significance threshold of $P = 1\text{x}10^{-5}$.
